# Modulating the extension of axillary lymphadenectomy for early stage breast cancer

**Published:** 2016

**Authors:** M Plesca, C Bordea, B El Houcheimi, E Ichim, A Blidaru

**Affiliations:** * 2nd Department Surgical Oncology, “Prof. Dr. Alexandru Trestioreanu” Oncology Institute, Bucharest, Romania

**Keywords:** breast cancers, sentinel lymph node, axillary lymphadenectomy

## Abstract

Axillary lymph node evaluation remains essential in breast cancer surgery, first as a prognostic factor, because it indicates the degree of dissemination of the disease to the main lymphatic drainage basin of the breast, and, on the other hand, as an element of preventing the local relapse. In the era of the sentinel lymph node, complete axillary lymphadenectomy, considered valuable until recently, but as therapeutic and diagnostic, has become an intervention performed increasingly rare in selected cases. Axillary lymphatic tissue resections are accompanied by morbidity (lymphedema, paresthesia, limitations of arm movement) and symptom magnitude is proportional to the extension of the intervention. For this reason, a solution to avoid these kinds of complications was looked for. Since Gould, in 1960, who mentioned cancer parotid and continuing with Cabanas, Morton, or Veronesi, many surgeons have contributed to the development of safe techniques with which the multidisciplinary team involved in the surgical treatment for breast cancer could perform a safe oncological intervention and at the same time could conserve the healthy tissue, thus limiting morbidity. To achieve this standard, axillary lymphadenectomy has passed through several stages, from over radical interventions that followed the Halsted era, in which, besides axillary lymph nodes, the internal mammary and jugulo-carotidian lymph nodes were excised, to the absence of axillary surgery and replacing it with radiation therapy.

## Introduction

At present, axillary lymphadenectomy is an essential component of surgery with a radical intention for breast cancer. If in the beginning, the axillary lymphadenectomy had the aim of curing and ensuring the local control, currently, it has become a prognostic and diagnostic instrument, which guides the therapeutic attitude. The extent of the dissection diminished much since William Halsted performed the first radical mastectomy in 1882 [**1**] and recorded variations of amplitude culminating with the highly radical interventions from the 1950s made by Urban, who excised the internal mammary chain lymph nodes [**2**], though less than 5% of the breast cancers disseminate this level, as his subsequent studies showed [**3**]. With the introduction of the sentinel node identification and biopsy technique, it reached the point where a single lymph node would be considered sufficient to provide a fair picture of the status of the entire lymphatic basin [**4**]. There is data from recently conducted clinical trials according to which the axillary dissection extension does not significantly influence the survival but ensures the local control and is useful in disease staging [**5**]. Even if the sentinel node was invaded, no statistically significant differences were noticed in the overall survival between patients who underwent axillary lymphadenectomy and those for whom only the sentinel node biopsy was performed. However, before excluding the axillary surgery for breast cancer, some conclusions are necessary from clinical trials that are currently underway, evaluating the safety and the risk-benefit balance in making such a decision [**6**].

## Material and Method

A descriptive study was conducted on 368 cases of early stage breast cancer (T1-T2N0M0) in which sentinel node identification and biopsy was performed in the Surgical Oncology Department of “Prof. Dr. Alexandru Trestioreanu” Oncology Institute in Bucharest, between 2003 and 2013. The aims of the study were to present the results and to identify the clinicopathological characteristics that make up a clinical case in which the modulation of the extension of axillary lymphadenectomy might be considered even with positive sentinel nodes. Imagery was suggestive of breast cancer in all cases. Some cases needed associated breast additional MRI to better determine the full extension of some suspicious tumors or to differentiate multifocality or multicentricity of primary lesions. Surgery consisted of conservative treatment or Madden modified radical mastectomy. When breast lesions did not have a clinical expression, ultrasound, or mammography was used to localize them before surgery. In some cases of conservative treatment, oncoplastic surgery techniques were implemented to obtain a better aesthetic result without compromising the oncological safety. Also, an immediate reconstruction of the breast mammary prosthesis or expander after mastectomy was performed when there was a congruence between the indication for this procedure and the patient’s desire. The sentinel node biopsy in breast cancer allows the evaluation, the sentinel node status being considered the mirror of the remaining regional lymph nodes. It also allows selective axillary surgery and reduces morbidity associated with radical surgery.

The procedure was performed by using a radioactive tracer (radioactive isotope technetium 99 attached to albumin). After injecting a tracer in the proximity of the tumor or an intradermal periareolar dose of 37 MBq and volume of 0.5 ml about 20 hours before surgery, lymphoscintigraphy was performed, which showed the site and number of the sentinel nodes, the tegumentar marking being done accordingly. The probe used to identify sentinel nodes was NeoProbe2000. Using this probe intraoperatively, the axillary point with the most intense radioactivity was identified by a skin incision, which was done superjacent. There have also been cases in which the intervention was achieved through a single incision for aesthetic reasons, conveniently placed to be able to practice both primary tumor excision with margins of safety and sentinel node biopsy. Depending on the positive status of the sentinel node, the axillary surgery continued, achieving a complete axillary lymphadenectomy.

### Results

The study included 368 cases, of which, 2 were males and 366 females with T1-T2 breast tumors N0 M0. At diagnosis, the youngest patient was 28 and the oldest was 77. In more than half of the cases, (53%) patients were older than 50 years. According to current TNM standard (7 edition AJCC), in terms of tumor size, there were lesions with a diameter between 0.5 and 4 cm. In 48% of the cases, the primary tumor was located in the external quadrants and 32% of the patients it was located in the internal quadrants. The tumor was located in the central quadrant in 20% of the cases. The result of the histopathology was invasive ductal carcinoma (79%), invasive lobular carcinoma (18%), and carcinoma in situ (3%). The immunohistochemical examination often showed a known fact, that breast cancer is stimulated by hormone secretion, 83% of the patients having tumors with estrogen and progesterone receptors [7]. Also the two male cases included in the study had tumors with a positive hormonal profile. A molecular classification of cases with hormonal status criteria receptors, HER2 + and Ki67 proliferation index were conducted as it follows: 68% luminal (HR +, HER2, Ki67 < 14%), 15% Luminal B (HR +, HER2 + / Ki67 > 14%), 12% HER2 + (HR-HER2 +), 5% triple negative (HER2, HR-).

Sentinel node biopsy was successful in all the cases. The tracer migrated in most cases (94% - 347 cases) to the axillary lymphatic basin, there were 12 cases in which lymphoscintigraphy showed drainage to multiple sites, both axillary and internal mammary. In 4% of the cases (14 patients), the sentinel node was identified in the internal mammary chain lymph nodes. In 2% of the cases, the other sites were intramammary (4 cases) or lateral pectoral (3 cases) lymph nodes described by Sappey or Sorgius [**8**]. No interpectoral or supraclavicular sentinel nodes were identified in the study group. There were cases in which several sentinel lymph nodes were identified during surgery, the highest case consisting of 3 nodes. In 77 patients, the histopathological result of the intraoperative sentinel node was positive. There were also 5 cases of false negative sentinel nodes, meaning that the final histological report invalidated the intraoperative exam. No invaded lymph nodes were found in 286 cases. Of the 77 patients with positive sentinel nodes, in 63% of the cases, the sentinel node was the only positive node. In 28 cases, about 37% of the cases, the sentinel node was positive with further positive nodes, as it follows: between 1-3 positive nodes were registered in 14 cases while in the other 14 cases, there were more than 3 positive nodes. The average number of lymph nodes excised in cases in which complete axillary lymphadenectomy was performed was of 14 nodes. Addressing the primary lesion of the breast was done by practicing the excisional biopsy of the tumor followed by additional mammary gland resection (conservative treatment) in 67% of the cases - 246 patients, in the remaining 122 cases, surgery consisted of Madden modified radical mastectomy.

**Fig. 1:**
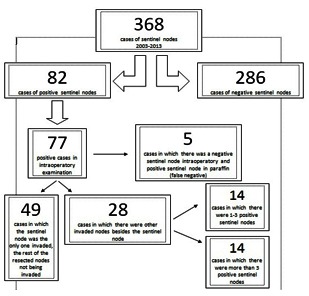
**Fig. 1**Overall cases

**Fig. 2:**
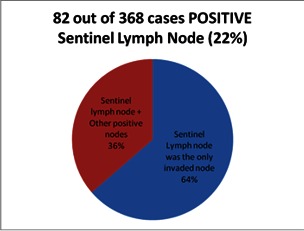
**Fig. 2**Sentinel lymph node invasion

## Discussion

For a long time, axillary lymphadenectomy for breast cancer was considered standard but currently there have been some clinical trials and ongoing studies indicating the futility of this intervention, at least in the selected cases. The most important studies, led by Giuliano - ZOO11 ACOSOG (USA) and 23-1 IBCSG led by Veronesi (Italy), which showed that the axillary lymphadenectomy can be avoided in patients with less advanced stage breast cancer and positive sentinel nodes, and that there is no significant differences in terms of survival, both overall and disease free between these patients and those who underwent complete axillary lymphadenectomy [**9**,**10**]. This hypothesis was confirmed by the EORTC-AMAROS trial that found no difference between patients with breast cancer T1-T2 and positive sentinel node, who underwent lymphadenectomy and those who received only adjuvant radiation therapy, with obvious benefits in limiting the morbidity associated with axillary dissection [**11**].

Regarding the cases with positive sentinel nodes, noteworthy is the number of cases with lymph node invasion importance: 14 cases that have been invaded with more than 3 lymph nodes including the sentinel node. These cases are, in terms of the findings of the mentioned trials, the only ones in which the axillary lymphadenectomy was helpful. So, for 82% of the cases, the axillary lymphadenectomy could have been avoided, according to the same authors. However, there are some controversies surrounding these studies. First, for the highest level of confidence, a study must be randomized and blinded [**12**]. Regarding ACOSOG Z0011, there are question marks regarding the way that was achieved; the doctors who performed the radiation therapy for highly suspicions cases (aggressive histopathological profile, young age, ultrasound suspicious axillary lymph nodes) were suspected of drawing radiotherapy tangents to include a larger field for patients who did not complete the axillary lymphadenectomy [**13**]. There is also a number of published articles stating that there were flaws in the patients selection process; the sentinel node biopsy alone was practiced only in cases in which the clinical and imagistic context indicated a lower probability of invasion [**14**]. If the issue of local control of the disease in patients with sentinel node positive radiotherapy provided a solution (AMAROS-EORTC), a controversy over the selection of patients in the clinical picture will find its answer in the results of the SOUND trial (Sentinel node vs. Observation after axillary ultrasound) coordinated by IEO Milan [**15**]. This randomized multicentre trial aimed to divide breast cancer patients into two arms, one arm with sentinel node biopsy, and one arm with axillary ultrasound evaluation without a surgical approach if no suspicious lymph nodes are detected. The assumption is that for early stages of breast cancer, where there is no suspicion both clinical and radiological, the axillary surgery being worthless from the therapeutic standpoint [**16**].

## Conclusion

Today, axillary lymphadenectomy has remained an essential component of breast cancer surgery. The complete axillary lymphadenectomy is necessary for cases in which mastectomy is performed without postoperative irradiation. A full axillary lymphadenectomy is mandatory for cases with 3 or more positive nodes, for diagnosis and local control of the disease. In less advanced stages of breast cancer, sentinel node biopsy can be considered sufficient to provide a fair picture of the entire lymphatic basin status and can establish the indication for selective lymphadenectomy. By excluding the axillary surgery for breast cancer, conclusions from the currently ongoing clinical trials to evaluate the safety and benefit/ risk of such a decision are required. The results of this study confirmed the data reported in the field from highly experienced centers.

### Acknowledgements and Funding

This work received financial support through the project entitled “CERO- Career profile: Romanian Researcher”, grant number POSDRU/159/1.5/S/135760, cofinanced by the European Social Fund for Sectoral Operational Programme Human Resources Development 2007-2013.
